# Determinants of ultrasound-guided reduction failure and pathological lead points in pediatric intussusception

**DOI:** 10.1007/s00383-026-06315-8

**Published:** 2026-02-16

**Authors:** Yannick Braun, Henning C. Fiegel, Udo Rolle, Till-Martin Theilen

**Affiliations:** 1 Department of Pediatric Surgery and Pediatric Urology, Goethe University Frankfurt, University Medicine, Frankfurt, Germany; 2 Universitätsklinikum Frankfurt, Theodor-Stern-Kai 7, 60590 Frankfurt am Main, Germany

**Keywords:** Intussusception, Hydrostatic reduction, Ultrasound-guided enema, Pathological lead point, Predictive factors

## Abstract

**Purpose:**

Intussusception is a leading cause of acute intestinal obstruction in children. Ultrasound-guided hydrostatic saline enema (USGSE) is widely accepted as first-line non-surgical management, but predictors of reduction failure and pathological lead points (PLPs) are not well defined.

**Methods:**

We retrospectively reviewed pediatric patients (< 18 years) with ileocolic intussusception treated between 2012 and 2022. Clinical variables included symptom duration, vomiting, bloody stools, and age. Univariable and multivariable logistic regression analyses were used to identify predictors of failed USGSE and PLPs.

**Results:**

Eighty-nine patients (93 episodes) were analyzed; 97.85% underwent USGSE as initial treatment. Overall reduction success was 76.92% (70/91) and 90.28% (65/72) in patients without PLPs, with no complications. Symptom duration > 24 h was associated with failed USGSE (OR 4.29, *p* = 0.0052). After excluding PLP cases, predictors of failure included symptom duration > 24 h (OR 13.97, *p* = 0.0059), bloody stools (OR 6.83, *p* = 0.0245), and younger age (*p* = 0.0094). PLPs were identified in 18 patients (20.2%), most commonly Meckel’s diverticulum. Failed USGSE was the sole independent predictor of a PLP (OR 107.26, *p* < 0.0001).

**Conclusion:**

USGSE is safe and highly effective for pediatric ileocolic intussusception. Prolonged symptoms and bloody stools predict reduction failure, while failed USGSE strongly indicates an underlying PLP, supporting prompt intervention and surgical evaluation when reduction is unsuccessful.

**Supplementary Information:**

The online version contains supplementary material available at 10.1007/s00383-026-06315-8.

## Introduction

 Intussusception is a potentially life-threatening condition in which a segment of the intestine telescopes into an adjacent distal segment, resulting in bowel obstruction and impaired mesenteric blood flow [1–3]. The consequent venous congestion, edema, and resulting ischemia may progress to necrosis and perforation if not treated adequately. Clinically, intussusception may present with a wide spectrum of symptoms ranging from intermittent abdominal pain and vomiting to diarrhea, lethargy, irritability, and hematochezia. In infants and young children, the classic triad of colicky abdominal pain, vomiting, and “currant jelly” stools—although well recognized—is present in only a minority of cases, making early diagnosis challenging [4, 5].

The condition predominantly affects infants and young children between 6 months and 2 years of age, with an estimated incidence of approximately 20–50 cases per 100,000 infants per year in large cross-sectional studies, and a notable male predominance [6–8]. In contrast, adult intussusception is uncommon, with an incidence of approximately 2–3 cases per million per year, and typically occurs secondary to a pathological lead point (PLP) such as a tumor, polyp, or inflammatory lesion [9, 10]. In children, however, the etiology is usually idiopathic, most commonly associated with hypertrophy of Peyer’s patches or other lymphoid tissue following viral infections, most prominently adenovirus [11–13]. PLPs in pediatric populations include Meckel’s diverticulum, intestinal duplication, polyps, and tumors [14, 15]. Small bowel–small bowel intussusceptions are often transient and self-resolving in the absence of a pathological lead point, whereas ileocolic intussusception generally requires urgent reduction due to its rapid progression and higher risk of ischemic injury.

Diagnostic confirmation is typically achieved through imaging, with ultrasound being the modality of choice in children due to its high sensitivity, specificity, and lack of radiation exposure [16]. The characteristic “target” or “doughnut” sign on transverse imaging is the classical radiological correlate [17]. Once confirmed, non-surgical reduction is the preferred initial management approach. Both pneumatic (air) and hydrostatic (saline) enemas are widely utilized, with air enema being more common in the United States and the United Kingdom [18–20]. Hydrostatic ultrasound-guided saline enema (USGSE) has gained attention in many centers as it avoids radiation and allows real-time assessment of reduction. Reported success rates for non-surgical reduction range from 65% to 95%, depending on the physician’s experience and patient selection [21, 22].

Despite its overall effectiveness, a proportion of cases fail non-surgical reduction and require surgical intervention. Failure may be related to delayed presentation, bowel necrosis, or the presence of a PLP. Early identification of patients at risk of failed USGSE or underlying pathology is therefore essential to optimize management strategies, minimize complications, and reduce unnecessary delays in surgical treatment.

This study aimed to identify clinical predictors associated with unsuccessful USGSE reduction and to determine factors suggestive of a pathological lead point in pediatric ileocolic intussusception.

## Methods

### Study design and patients

This retrospective observational single-center study focused on patients below 18 years of age with a final diagnosis of ileocolic intussusception treated in the pediatric surgery center of the University Hospital Frankfurt between 2012 and 2022. Inclusion criteria were defined as follows: (1) Age under 18 years; (2) Interventional procedure (USGSE, surgery, or both). Data concerning each patient’s initial presentation and clinical course were assessed through the in-hospital medical record database. Extracted parameters were: Age at presentation; Sex; Duration of symptoms (< 24 h or > 24 h); Vomiting; Bloody stools; Number of USGSE attempts during the same hospital stay; Complications during USGSE; Success of USGSE; Pathological records.

Analyses were conducted at both the case-based and patient-based levels. At the case-based level, each presentation was treated as an independent event, whereas at the patient-based level, individuals with multiple presentations were considered as a single event based on the final diagnosis and final clinical assessment (Failure of USGSE; Presence of a PLP).

During the case-based analysis, USGSE reduction was deemed successful if, during the acute hospital stay, surgical reduction of intussusception was not necessary, even if USGSE was carried out repetitively or a PLP was later identified in the same patient during a subsequent stay.

### Outcomes

Our primary outcomes were the failure of the USGSE reduction of intussusception and the presence of PLPs in patients with intussusception. Secondary outcomes were the incidence of complications during USGSE and the number of recurrences during the same hospital stay.

### Statistical analyses

All the statistical analyses were performed using R (v.4.3.2, R Foundation for Statistical Computing, Vienna, Austria). Differences were considered significant if *p* < 0.05. Continuous data between two groups were tested using a two-sided, two-sample t-test for parametric data. Categorical variables between two groups were tested using Fisher’s exact test. Two multiple binary logistic regression models were constructed: One to analyze the influence of clinical parameters on the failure of USGSE (Sex, Symptom duration, Bloody stools, Vomiting, Age), and the other to analyze the influence of clinical parameters on the presence of USGSE (Sex, Symptom duration, Bloody stools, Vomiting, Age, Failure of USGSE).

## Results

### Patients and clinical course

**Fig. 1:**
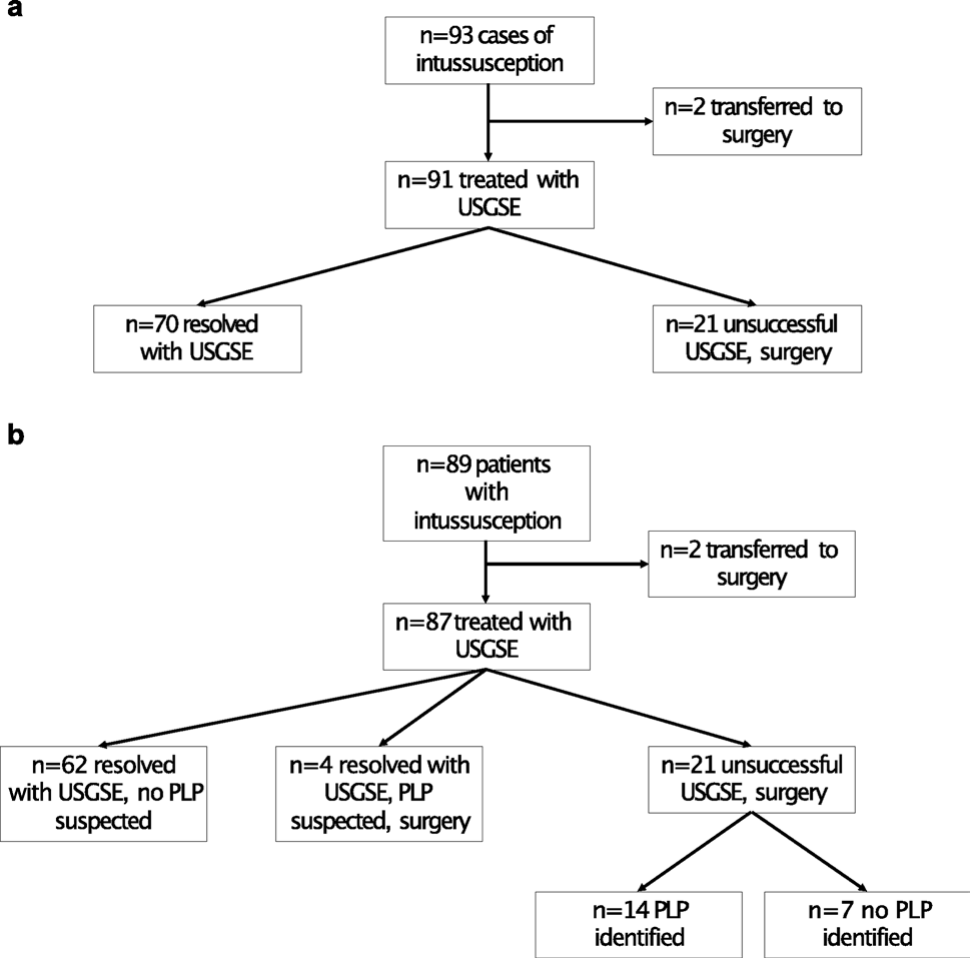
**a** Flowchart depicting the clinical course during the case-based analysis; **b** Flowchart depicting the clinical course during the patient-based analysis

A total of 89 patients with 93 episodes of ileocolic intussusception presented to our clinic during the study period. Clinical data regarding all patients are summarized in Table 1. USGSE was the primary treatment chosen in 91 of 93 (97.85%) cases or 87 of 89 patients (97.75%) (Fig. 1a). Both other patients were transferred directly to surgery as symptoms, consistent with prolonged ischemia and deteriorated clinical status, were present at the time of referral. These cases showed intussusception with intestinal necrosis during surgery.

USGSE was successful in the reduction of intussusception in 70 of 91 cases (76.92%). No complications (perforation, sepsis, adverse events of sedation) occurred during the performance of USGSE. 26 of 70 cases (37.14%) successfully treated with USGSE required repetitive enema due to short-term recurrence of intussusception during the same hospital stay. In these cases, the median amount of recurrences was one; the maximum amount of recurrences was four.

In 21 cases, patients required surgery after failed enema reduction. In 14 of 21 (66.67%) cases, a PLP was identified during surgery. PLPs were Meckel´s diverticulum (11), intestinal polyps (2), and abdominal B-cell lymphoma (1).

**Table 1:** Patient characteristics and presenting complaints

All patients (*n* = 89)	
Male sex, n (%)	57 (64.04)
Age at presentation (months), mean (SD)	28.04 (27.58)
Symptoms > 24 h, n (%)	36 (40.45)
Presence of bloody stools, n (%)	26 (29.21)
Presence of vomiting, n (%)	50 (56.18)
Recurrence during hospital stay, n (%)	26 (29.21)

*SD* Standard deviation

Four patients presented with long-term recurrences (> 4 weeks after first presentation) of intussusception after successful USGSE. In three of these cases, USGSE resolved the intussusception, but in one case, a pathological lead point was suspected, and subsequently identified and treated during surgery.

In total, four patients with successful primary enema reduction of intussusception received an operative procedure because of suspicion of a pathological lead point during USGSE (Fig. 1b). A PLP was identified in all cases (2 Meckel´s diverticula, 1 abdominal B-cell lymphoma, 1 intestinal polyp).

### Predictors of the failure of USGSE

To identify predictors for the failure of USGSE, we analyzed the extracted clinical parameters and their association with non-successful reduction. Only the duration of symptoms exceeding 24 h significantly correlated with an increased risk of USGSE failure (OR 4.29 (1.39–14.46), *p* = 0.0052). Sex, presence of bloody stools, presence of vomiting, and age were not associated with non-successful reduction of intussusception (Table 2).

**Table 2:** Correlation between clinical data and USGSE success

USGSE cases (*n* = 91)	Success, (*n* = 70)	Failure (*n* = 21)	*p*-value	OR (95%-CI)
Male sex, n (%)	47 (67.14)	13 (61.9)	0.7935*	1.25 (0.39–3.83)
Duration of symptoms > 24 h, n (%)	22 (31.43)	14 (66.67)	0.0052*	*4.29 (1.39–14.46)*
Presence of bloody stools, n (%)	18 (25.71)	7 (33.34)	0.5791*	1.44 (0.42–4.58)
Presence of vomiting, n (%)	38 (54.29)	13 (61.9)	0.6208*	1.36 (0.46–4.31)
Age at diagnosis in months, mean (SD)	28.12 (24.2)	22.67 (27.1)	0.4127^#^	

*Fisher´s exact test

^#^Students t-test

*SD* Standard deviation, 95% *CI* (95% Confidence Interval), level of significance p<0.05

We hypothesized that the high number of patients with a PLP in our cohort may affect the results of the previous analysis because of the increased rate of non-successful enema reduction expected in these patients. Therefore, we opted to exclude these patients and repeat the analysis. After exclusion of patients with a PLP present (18 of 89 patients, 20.22%), a total of 72 presentations with intussusception remained (Table 3). USGSE was successful in 65 of 72 cases (90.28%). In these cases, duration of symptoms > 24 h (OR 13.97 (1.54–679.6), *p* = 0.0059), presence of bloody stools (OR 6.83 (1.01–78.01), *p* = 0.0245), and patient age (*p* = 0.0094) were significantly associated with the failure of USGSE. Gender and presence of vomiting did not correlate with non-successful reduction with USGSE. On the other hand, no clinical parameter influenced the success of USGSE in cases with a pathological lead point present (Supplementary Table 1). When we constructed a binomial logistic regression model to predict the failure of USGSE with the extracted clinical parameters, the duration of symptoms was the only clinical parameter significantly associated during this multivariable analysis (OR 35.81(2.67–2534.03.67.03), *p* = 0.0291) (Supplementary Table 2 and Table 3).

**Table 3:** Correlation between clinical data and USGSE success in patients without a PLP

USGSE attempts without pathological lead point (*n* = 72)	Success (*n* = 65)	Failure (*n* = 7)	*p*-value	OR (95%-CI)
Male sex, n (%)	43 (66.15)	5 (71.43)	1*	0.78 (0.06–5.28)
Duration of symptoms > 24 h, n (%)	19 (29.23)	6 (85.71)	0.0059*	*13.97 (1.54–679.6)*
Presence of bloody stools, n (%)	17 (26.15)	5 (71.43)	0.0245*	*6.83 (1.01–78.01)*
Presence of vomiting, n (%)	35 (53.85)	5 (71.43)	0.4511*	2.12 (0.32–23.81)
Age at diagnosis in months, mean (SD)	26.71 (24.2)	11.29 (10.9)	*0.0094* ^#^	

*Fisher´s exact test

^#^Students t-test

*SD* Standard deviation, 95% *CI* (95% Confidence Interval),  level of significance p<0.05

### Predictors of pathological lead points

In the next step, we wanted to identify predictors of PLPs in patients with intussusception. During univariable analysis, only the failure of a USGSE significantly correlated with the presence of a PLP (OR 28.84(6.84–156.9), *p* < 0.0001). Of note, a substantial percentage of patients (4 of 18, 22.22%) with a PLP were successfully treated during initial presentation with USGSE. None of the other extracted clinical parameters showed an association with the presence of a PLP (Table 4). When we constructed a binomial logistic regression model for the presence of a PLP against the failure of USGSE was the only significant factor in this multivariable analysis (OR 107.261(16.817–1516.628.817.628), *p* < 0.0001) (Supplementary Table 3).

**Table 4:** Correlation between clinical data and the presence of a PLP

Patients treated with USGSE (*n* = 87)	PLP (*n* = 18)	No PLP (*n* = 69)	*p*-value	OR (95%-CI)
Male sex, n (%)	11 (61.11)	45 (65.22)	0.7863*	1.19 (0.34–3.89)
Duration of symptoms > 24 h, n (%)	10 (55.56)	24 (34.78)	0.1738*	2.32 (0.72–7.77)
Presence of bloody stools, n (%)	3 (16.67)	22(31.88)	0.2535*	0.43 (0.07–1.75)
Presence of vomiting, n (%)	11 (61.11)	38 (55.07)	0.7911*	1.28 (0.39–4.38)
Age at diagnosis in months, mean (SD)	33.67 (29.6)	24.14 (23.1)	0.2179^#^	
Failed USGSE, n (%)	14 (77.78)	7 (10.14)	< 0.0001*	*28.84 (6.84–156.9)*

*Fisher´s exact test

^#^Students t-test

*SD* Standard deviation, * 95% CI*(95% Confidence Interval),  level of significance p<0.05

## Discussion

Intussusception remains one of the most frequent causes of acute abdominal pain and bowel obstruction in infants and young children [1, 2]. Prompt diagnosis and management are crucial to prevent serious complications such as bowel ischemia, necrosis, and perforation. The introduction of ultrasound-guided hydrostatic reduction techniques has transformed the non-surgical management of intussusception, offering a safe, effective, and radiation-free alternative to fluoroscopic air enema [23, 24]. In our cohort, ultrasound-guided saline enema (USGSE) demonstrated excellent safety and efficacy, with no complications observed during the procedure, even in patients presenting with a prolonged symptom duration of up to 72 h. This supports growing evidence that USGSE can be safely attempted in stable patients, provided that appropriate clinical and supportive measures are implemented.

The absence of complications using USGSE in our study, even after repeated USGSE in recurrent intussusception, supports its role as a first-line management option. Adequate sedation and analgesia are essential prerequisites for procedural success, as patient comfort minimizes involuntary straining and facilitates smooth hydrostatic pressure transmission. In addition, careful fluid and hemodynamic management contributes to intestinal perfusion stability, enhancing both safety and efficacy [25, 26].

Compared to air enema under fluoroscopic guidance, USGSE offers the distinct advantage of real-time sonographic visualization of the intussusceptum and a potential PLP, without exposure to ionizing radiation [16]. Numerous comparative studies have demonstrated that ultrasound-guided hydrostatic reduction is non-inferior to pneumatic reduction with respect to success rates and complication profiles [21, 27–29]. Our findings align with these data, reinforcing that USGSE should be considered the preferred approach, particularly in pediatric populations, where minimizing radiation exposure is of paramount importance.

A unique advantage of USGSE is its diagnostic potential during the reduction process. As highlighted by our results, ultrasound not only allows direct observation of the reduction but also facilitates the identification of potential pathological lead points (PLPs) in real time. In our series, only one patient who experienced recurrence after an initially successful reduction was later found to have an intestinal polyp as the pathological lead point, which was subsequently resected. In three other patients, a PLP was suspected during initial treatment with a saline enema. All three patients underwent scheduled procedures that confirmed the presence of the suspected PLP. This underscores the additional diagnostic capabilities of a USGSE in pediatric intussusception.

Our analysis also identified several factors influencing the likelihood of successful reduction. Consistent with previous literature, a longer duration of symptoms prior to presentation correlated with a higher risk of reduction failure [30, 31]. This relationship likely reflects progressive mucosal edema, venous congestion, and early ischemic changes that reduce the pliability of the bowel wall and hinder reduction. Similarly, the presence of bloody stools—a clinical marker of mucosal injury—was significantly associated with unsuccessful reduction in patients without an underlying PLP. These findings reaffirm the importance of early diagnosis and timely referral for non-surgical reduction to optimize outcomes.

Age has been reported as a predictor of failed non-surgical reduction [32, 33]. In our cohort, age did not significantly influence reduction success when all patients were included. However, after excluding cases with PLPs, younger age emerged as a predictor of failed USGSE. This pattern suggests that the role of age as a predictive factor may depend on underlying pathophysiology and sample composition. Symptom duration exceeding 24 h was the only clinical factor associated with an increased risk of failure of USGSE in our multivariable analysis. Highlighting the importance of an early diagnostic assessment in children with suspected intussusception.

The identification of a pathological lead point remains clinically crucial, as its presence necessitates further surgical or endoscopic management. In our cohort, failed USGSE was the single most significant predictor of a PLP. This finding aligns with other studies showing that non-surgical reduction failure often indicates a structural cause, such as a polyp, Meckel’s diverticulum, or duplication cyst, preventing spontaneous or hydrostatic reduction [15, 34, 35]. Since patients with failed USGSE typically proceed to surgery, this association may be somewhat amplified, as operative exploration inherently increases PLP detection. Conversely, PLPs may remain underdiagnosed in cases of successful reduction if not carefully evaluated during USGSE, follow-up imaging, or subsequent presentations. Nevertheless, the overall prevalence of PLPs in pediatric intussusception remains low, and our results support that the risk of missed diagnosis following successful USGSE is minimal when imaging is appropriately performed.

From a clinical perspective, these findings have several implications. First, USGSE should continue to be adopted as the primary non-surgical treatment modality for intussusception, given its high success rate, excellent safety profile, and additional diagnostic capabilities. Second, the presence of prolonged symptoms or bloody stools should alert clinicians to a higher risk of failed reduction, prompting consideration of early surgical consultation if hydrostatic attempts are unsuccessful. Finally, every failed USGSE should trigger a systematic intraoperative search for a pathological lead point, as its identification and treatment are essential for preventing recurrence.

Despite its strengths, our study has several limitations. The single-center, retrospective design and moderate sample size may limit generalizability. Future multicenter prospective studies with standardized protocols and long-term follow-up are warranted to validate these findings and refine predictive models for both USGSE success and PLP identification.

## Conclusion

In conclusion, our data reaffirm that ultrasound-guided hydrostatic saline enema is a safe, effective, and reproducible first-line treatment for pediatric intussusception, offering real-time diagnostic insight and eliminating radiation exposure. Failed USGSE remains the strongest predictor of a pathological lead point and should always prompt comprehensive surgical evaluation. With appropriate procedural technique, sedation, and patient monitoring, USGSE represents the optimal non-surgical approach for managing ileocolic intussusception in children.

## Supplementary Information

Below is the link to the electronic supplementary material.

Supplementary material 1 (DOCX 90 kb)

## Data Availability

Raw data files are available upon request to the corresponding author.
