# Supplementary material for: Determinants of ultrasound-guided reduction failure and pathological lead points in pediatric intussusception
Source: Pediatr Surg Int. 2026 Feb 16;42(1):91. doi: 10.1007/s00383-026-06315-8 (PMC12909316; doi:10.1007/s00383-026-06315-8)
Supplement: Supplementary file 1 — Supplementary material 1 (DOCX 90 kb) [file 383_2026_6315_MOESM1_ESM.docx]

Supplementary Table 1

Correlation of clinical data and USGSE success in patients with PLP

| **USGSE attempts without pathological lead point (n=19)** | **Success**  **(n=5)** | **Failure**  **(n=14)** | **p-Value** | **OR (95%-CI)** |
| --- | --- | --- | --- | --- |
| **Male sex, n (%)** | 4 (80) | 8 (57.14) | 0.6027* | 2.84 (0.20-171.7) |
| **Duration of symptoms >24h, n (%)** | 3 (60) | 8 (57.14) | 0.3378*** | 0.27 (0.01-3.69) |
| **Presence of bloody stools, n (%)** | 1 (20) | 2 (14.29) | 1* | 0.68 (0.03-49.06) |
| **Presence of vomiting, n (%)** | 3 (60) | 8 (57.14) | 1* | 0.89 (0.06-10.71) |
| **Age at diagnosis in months, mean (SD)** | 28.43 (31.1) | 46.4 (17.6) | 0.1407^#^ |  |

Supplementary Table 2

Binary logistic regression analysis for the failure of USGSE

**OR 2.5 % 97.5 % p-value**

(Intercept) 0.01 0.00 0.66 0.0623
**Male Sex** 1.25 0.13 10.71 0.8343

**Duration of symptoms >24h** 35.81 2.67 2534.03 *0.0291*

**Presence of bloody stools** 14.95 0.80 870.27 0.1084

**Presence of vomiting** 0.12 0.00 2.08 0.1740

**Age at diagnosis in months**  1.01 0.91 1.09 0.8624

Supplementary Table 3

Binomial logistic regression analysis for the presence of a PLP

**OR 2.5 % 97.5 % p-value**

(Intercept) 0.01 0.00 0.31 0.0165

**Male Sex**  0.76 0.12 4.02 0.7521

**Duration of symptoms >24h**  1.71 0.33 9.35 0.5181

**Presence of bloody stools** 0.11 0.01 1.09 0.0771

**Presence of vomiting** 3.05 0.52 25.65 0.2430

**Age at diagnosis in months**  1.03 1.00 1.08 0.0734

**Failure of USGSE**  107.26 16.82 1516.63 *<0.0001*
